# Low Carbohydrate Diets in Cancer Therapeutics: Current Evidence

**DOI:** 10.3389/fnut.2021.662952

**Published:** 2021-11-25

**Authors:** Christopher Haskins, Justin Cohen, Rupesh Kotecha, Adeel Kaiser

**Affiliations:** ^1^Department of Radiation Oncology, University of Maryland Medical Center, Baltimore, MD, United States; ^2^Miami Cancer Institute, Baptist Health, Miami, FL, United States; ^3^Herbert Wertheim College of Medicine, Florida International University, Miami, FL, United States

**Keywords:** cancer, metabolism, ketogenic diet (KD), carbohydrate restriction, radiation, chemotherapy, immunotherapy

## Abstract

Low carbohydrate diets have a promising mechanistic rationale in the treatment of cancer with favorable preclinical data. The strongest data suggest synergistic effects of dietary interventions with traditional cancer therapies. Recent prospective clinical trials suggest that low carbohydrate diets are safely and feasibly added within a busy oncology clinic, with hopeful additive effects in treatment enhancement.

## Introduction

Cancer continues as the second leading cause of death across the United States, with an estimated 1.9 million new cancer cases expected in 2021 (1, 2). Cancer therapeutics also continues to progress. According to the American Cancer Society statistics, as of 2018 the solid tumor death rate declined to 128 per 100,000 age adjusted persons from a peak of 175 in 1991 (3). While this progress should be lauded, the solid tumor death rate spanning back to 1930 was 190 per 100,000 age adjusted persons—a minimal reduction in 60 years. Furthermore, cancer treatment can accelerate aging, increasing the risk for comorbid conditions such as cardiovascular disease, diabetes, and osteoporosis (4, 5). As such, nutrition is of increasing interest in the oncology clinic, not only to decrease the risks of treatment sequelae, but also possibly to both prevent and treat disease. It is estimated that 80,000 cancer cases per year could be prevented with an adequate diet alone (6–8).

In 1930, Otto Warburg published his seminal work revealing an altered metabolism in solid tumors. Normal cells in high oxygen environments generate most of their cellular energy through aerobic respiration, processing glucose through the mitochondria to produce ATP. Tumor cells, on the other hand, shunt most glucose to lactate even in the presence of oxygen, called aerobic glycolysis, or more commonly the “Warburg effect” (9). In a recent thought provoking discussion, Vander Heiden et al. proposed the less efficient metabolism is adapted by tumor cells for the purpose of biomass production (10). Leveraging this metabolic distinction is now the basis for nutrition in oncologic research. Multiple dietary strategies have been employed, including the ketogenic diet (KD), caloric restriction (CR), and intermittent fasting.

The ketogenic diet, originally developed for the treatment of intractable epilepsy, aims to restrict carbohydrate and protein intake (11). Under low carbohydrate conditions, generally achieved through minimal carbohydrate intake (<30 g), the liver produces beta-hydroxybutyrate (BHB) from fatty acids. Ketosis is also often achieved with some caloric restriction and fasting regimens, as carbohydrates are subsequently also restricted below these levels. BHB is a ketone body uniquely suited to bypass Warburg metabolism given it cannot be metabolized back to glucose. It may therefore minimize tumor production while supplying adequate energy to normal tissues, particularly the brain. Indeed, some preclinical studies indicate some treatment efficacy with a KD therapy alone.

On the other hand, a recent meta-analysis of studies in mice showed that a KD is unlikely to cure any disease outright (12). Furthermore, tumors have shown to be metabolically flexible by shifting their energy source in response to a change in the nutrient environment (13, 14). Still, multiple preclinical and clinical studies do show some efficacy of intervention by synergizing with current cancer therapies.

## Carbohydrate Restriction and Cancer Care

### Systemic Therapy

#### Immunotherapy

Immunotherapy has become a mainstay of cancer therapy. Clinical trials have now demonstrated long-term increases in overall survival using antibodies targeting cytotoxic T-lymphocyte-associated protein 4 (CTLA-4), programmed cell death 1 (PD-1), or programmed death–ligand 1 (PD-L1), in comparison to conventional chemotherapeutic agents (15–17). However, not all patients respond to immunotherapy, even when they share the same histology. Although causes for differential responses are likely multifactorial, host microbiota has been implicated as a significant factor. In fact, a recently completed phase 1 feasibility study showed that fecal microbiota transplant could convert anti-PD-1 non-responders to responders (18). These preliminary results have now sparked a number of ongoing clinical trials of fecal transplant in immunotherapy non-responders for a variety of cancer types (NCT04729322, NCT03353402).

Since diet is a critical component of microbiota composition (19), researchers from France recently reported preclinical data bridging nutrition and cancer immunosurveillance (20). They found that 3-hydroxybutyrate (3HB) supplementation or a KD could induce response to immunotherapy when standard diet conditions did not permit one. Mechanistically, they noted that *E. massiliensis* was more abundant in the stools of ketogenic fed mice compared to controls. This correlates well with human data from the 1,000 patient UK PREDICT-1 study showing a positive correlation between *E. massiliensis* and ketone levels (*p* < 0.05) (21). 3HB also increased CD8+ T cells stimulated by immune checkpoint blockade. Interestingly, they also found that effect of 3HB was enhanced when it was given intermittently. This would likely permit easier application in the clinic through intermittent fasting techniques.

#### Chemotherapy

Although immunotherapy has garnered significant attention, chemotherapy remains the core of systemic cancer treatments. Researchers from University of Iowa demonstrated that a ketogenic diet may enhance responses to chemoradiation in animal models of lung cancer (22). As a result of these and other such preclinical studies, several ongoing trials are now examining the integration of ketogenic diets with conventional chemotherapy agents. The KETOLUNG (NCT01419587) and KETOPAN (NCT01419483) trials based out of University of Iowa, aimed to examine the feasibility of the ketogenic diet with chemotherapy and radiation for lung and pancreas cancers, respectively, but were closed due to poor accrual. Similarly, the KETO-CARE trial (NCT03535701) is being conducted by researchers from Ohio State University in advanced breast cancer patients receiving chemotherapy. A separate trial from China aims to examine whether a ketogenic diet can enhance the response to irinotecan therapy in patients with recurrent or metastatic breast cancer (23).

In 2020, the randomized, phase 2 DIRECT trial of stage II/III breast cancer patients undergoing neoadjuvant chemotherapy demonstrated several positive findings with the incorporation of a fasting mimicking diet (24). The fasting mimicking diet restricts calories and as such, simultaneously restricts both carbohydrates and protein. A per-protocol analysis of patients who were compliant with the diet in at least half of their chemotherapy cycles revealed a significant increase in grade 4/5 Miller and Payne pathological response rates (OR 3.194, *p* = 0.031). Moreover, the more cycles completed with the fasting mimicking diet, the greater the response to therapy (*p* = 0.035). This effect was likely mediated through ketosis with 93% of compliant patients demonstrating ketone bodies in comparison to only 8% of non-compliant patients. The fasting mimicking diet is currently being examined in trials involving prostate cancer (NCT04292041), lung cancer (NCT03700437), and in patients with any advanced cancers undergoing chemotherapy (NCT04027478).

Additionally, a ketogenic diet is being investigated as a potential strategy to decrease side effects of the anti-cancer drugs. In 2008, researchers from the University of Southern California showed that ketosis from short-term, 48–60 h fasting provided protection to mice, but not to injected neuroblastoma cells, against high dose etoposide chemotherapy (25). Despite the high concentration of etoposide, which was three times the upper limit used in humans, 96% of mice survived with fasting while only 34% survived without it. These results prompted the initiation of a fasting dose-escalation study for patients undergoing chemotherapy (26). Twenty patients completed the study in cohorts of 24, 48, and 72 h fasts. COMET assay results indicated reduced DNA damage in leukocytes of patients who fasted >48 h (*p* = 0.08). Study authors concluded that fasting for 72 h around chemotherapy was safe and feasible.

Since inflammatory pathway activation has been shown to induce resistance to chemotherapy and metastatic proliferation, dietary strategies to reduce inflammation are being explored (27). Researchers at Thomas Jefferson University used a mouse model for triple negative breast cancer, an aggressive breast cancer variant, to demonstrate that chemotherapy induced inflammation could be reduced with a low carbohydrate approach involving caloric restriction (28).

Finally, a recent meta-analysis examined variables within six clinical trials. Two were surgical, three delivered chemotherapy, and one ADT. Two-hundred twenty-two patients comprised the trials in total, with 153 completing the trials (79 low-carbohydrate, 74 general diet). The included studies varied significantly in design and reporting, with a very heterogeneous group of cancer patients that make any results unreliable. Overall, there was no evidence to support the beneficial effects of a low-carbohydrate diet in anti-tumor therapy (29).

#### Metabolic Therapy

The PI3K-Akt-mTOR pathway regulates a number of critical cellular functions including mRNA translation, autophagy and metabolic processes such as lipogenesis (30). Although abnormalities in this pathway have been implicated in breast, colorectal, hematologic and many other cancers, drugs targeting the pathway, such as PI3K inhibitors, have thus far proven clinically ineffective (31). Researchers from Cornell hypothesized that drug induced hyperinsulinemia may be limiting the therapeutic impact of these agents. They conducted a series of experiments using PI3K inhibitors in various mammalian tumor models. Study results demonstrated that resistance to PI3K inhibitors is mediated through reactivation of the PI3K-mTOR signaling pathway by insulin feedback (32). This preclinical data was consistent with clinical studies in which hyperglycemia was exacerbated in patients with insulin resistance, and resulted in discontinuation of PI3K inhibitor therapy. The group from Cornell went a step further and examined whether resistance to PI3K inhibitors could be suppressed through dietary or drug interventions limiting insulin feedback. These strategies included the ketogenic diet as well as sodium-glucose co-transporter 2 (SGLT2) inhibitor drugs used in diabetics. The addition of either intervention resulted in enhanced response to P13K inhibitors. Although clinical trials incorporating these strategies remain forthcoming, ongoing studies of PI3K inhibitors have restricted diabetic participants as a result of these findings (NCT01623349, NCT03213678).

#### Hormonal Therapy

Hormonal factors are implicated in carcinogenesis and consequently, are often targeted in cancer therapy. As an example, testosterone is highly implicated in prostate cancer growth and development (33). Anti-androgen drugs are therefore employed as anti-cancer therapies to lower testosterone levels. Although effective, these drugs induce hyperglycemia and hyperinsulinemia (34, 35), which increase steroidogenesis and androgen levels, and ultimately cause resistance to these medications (36, 37). There are two trials out of the University of Maryland (NCT03194516) and Cedars-Sinai (NCT03679260) currently analyzing carbohydrate restricted diets in the setting of men with prostate cancer on active surveillance to study progression prior to cancer therapy. As with prostate cancer, many patients with breast cancer undergo hormonal therapy as part of their treatment. Researchers from Vanderbilt are currently conducting a trial examining the feasibility of a 2-week ketogenic diet intervention with letrozole based hormonal therapy prior to surgery (NCT03962647). They hope to examine the effect of the diet on PI3K signaling.

### Radiation Therapy

Preclinical models suggest a potential synergistic effect of carbohydrate deprivation and radiation therapy. A study out of the University of Iowa treated mice with pancreatic cancer xenografts fed either a ketogenic chow or standard rodent chow, all treated with radiation (12Gy in 2Gy fractions). The mice on the 4:1 KD chow lived longer (*p* = 0.05) with slower tumor growth (*p* = 0.05) and decreased weight loss (38). Given this preclinical data, a prospective feasibility trial then enrolled two patients with Stage II/III pancreatic cancer and seven patients with stage III (non-operable) or IV (oligometastatic) non-small cell lung cancer (NSCLC) undergoing chemoradiation with the addition of the same 4:1 KD. The KD was poorly tolerated in this cohort with only 3 of the combined 9 patients completing the trial due mostly to poor compliance.

In a small German series, six patients consumed a self-administered KD during radiotherapy in a prospective manner (39). There were no diet related treatment toxicities and no negative effects on any blood parameters noted in the study. They too concluded the diet was safe and feasible. The same group proceeded with the KETOCOMP trial, analyzing the body composition changes of a cohort of 59 breast cancer patients undergoing radiation therapy. Twenty-nine received a ketogenic diet and 30 remained on their standard diet with no changes (40). The ketogenic diet caused initial water loss, with significant losses in body weight and fat mass, without substantial changes in fat free or skeletal muscle mass. The patients' quality of life remained stable with the dietary intervention.

In an intracranial bioluminescent mouse model of glioma, mice were fed a 4:1 (fat:protein+carbohydrate) KD vs. standard chow. When compared directly without additional treatment, the mice on the KD chow lived statistically longer, with a median survival of 28 days vs. 23 (*p* = 0.005). However, when the KD was combined with radiation, a synergistic effect occurred increasing the median survival significantly (*p* = 0.0001) and apparently curing nine out of eleven animals of their disease (41). To study glioblastoma multiforme (GBM) in the clinic, a retrospective analysis at the University of Pittsburgh analyzed 53 patients with GBM undergoing concurrent chemoradiation, six of whom were also on a ketogenic diet (42). The diet was well-tolerated with no grade III toxicities, no episodes of symptomatic hypoglycemia, lower average glucose even in the presence of steroids, and four out of six patients alive at a median follow-up of 14 months.

An experience from Cincinnati reviewed 29 patients with grade II-IV gliomas who were prescribed a modified Atkins diet (MAD), a modified low carbohydrate diet less restrictive than the classical KD (2:1 vs. 4:1) (43). These patients underwent radiation therapy plus or minus chemotherapy depending on grade. Of the 29 patients, 19 had GBM. All 29 patients achieved ketosis during the study period, defined in this study by 0.5 mmol/L, with 23/29 achieving 1 mmol/L. Only one patient stopped the diet early before the 6 week course of radiation. No grade III or grade IV toxicities were observed. In addition, the study analyzed an exploratory endpoint of pseudoprogression (PSP). One hypothesis for the mechanism of PSP is radiation sensitization. In this cohort, of the 19 patients with GBM, 11 (58%) developed PSP after combined treatment with MAD and chemoradiotherapy. The authors concluded the diet was safe and feasible, and they speculated that, given the percentage of patients with PSP were increased compared to historical controls, perhaps MAD provides a sensitizing effect to radiation therapy.

There are six ongoing trials open studying the effects of the KD in the treatment of GBM. Michigan State University is recruiting 16 patients with GBM for concurrent and adjuvant KD along with standard of care surgery, chemoradiation, and adjuvant chemotherapy (NCT01535911). UC Health Cincinnati (NCT03451799), Cedars Sinai Medical Center (NCT03451799), the Mid-Atlantic Epilepsy and Sleep Center (NCT02302235), Waikatu Hospital (NCT0430869), and the Guangzhao Medical University (NCT03160599) all have feasibility trials open analyzing KD in conjunction with standard of care therapies for GBM. The University of Liverpool recently completed a trial with using the KD in the adjuvant setting but has not yet reported results (NCT03075514).

### Surgical Oncology

The relationship between carbohydrate restriction and surgical oncology has not been investigated as rigorously. In fact, carbohydrate loading is now a central component of the enhanced recovery after surgery (ERAS) perioperative care pathways to mitigate the stress response to surgery (44). The stress response consists of complex neurohormonal and inflammatory responses that results in activation of the sympathetic nervous system, and secretion of cortisol, vasopressin and glucagon, leading to catabolism of skeletal muscle and insulin resistance (45). As insulin is an anabolic hormone, the induced insulin resistance promotes a catabolic state (46). Animal models have demonstrated a heightened stress response following trauma when in a fasting state, whereas a fed state blunted the stress response (47). Improved postoperative insulin sensitivity has been correlated with a decrease in length of hospital stay and postoperative major complications, therefore, the ERAS society recommends intake of clear fluids until 2 h prior to anesthesia induction (48). A large meta-analysis which included 1,976 patients demonstrated shorter hospital stay, shortened time to flatus passage, and increased postoperative insulin sensitivity, though it did not demonstrate a significant difference in the rate of postoperative complications (44).

While there appears to be a clinical benefit to preoperative carbohydrate loading regarding peri-operative metrics, a preoperative carbohydrate load may be counterproductive from an oncologic standpoint in the case of the surgical removal of malignancies. An undesired consequence of surgical manipulation is tumor seeding and cancer cells being pushed into circulation. Following excision, circulating tumor cells following excision are correlated with worse survival (49). Carbohydrate loading may promote an environment where these cells can become a viable metastatic focus. A phase III randomized trial was conducted in Norway comparing the effects of preoperative carbohydrate loading vs. a standard fasting preoperative protocol in patients with operable breast cancer. Thirty seven patients were allocated to the carbohydrate arm and 43 to the fasting arm, with 31 patients in the carbohydrate arm and 35 in the fasting arm receiving the allocated intervention. Results indicated that for patients with estrogen receptor (ER) positive breast cancer, 70% of fed patients had high proliferation (defined as an mitotic activity index ≥10) as compared to 30% in the fasting group (*p* = 0.038). The carbohydrate intervention (CI) group demonstrated an increase in serum insulin and c-peptide. Relapse free survival was improved for ER positive patients in the fasting group as compared to the fed group (97% vs. 71%, *p* = 0.012), though on Kaplan-Meir analysis this was demonstrated only in T2 tumors. While promising, these findings should be taken with caution given the relatively small number of patients and the low number of events (50). Additionally, though in the fed arm they were loaded with a carbohydrate diet, further analyses are required to determine whether the benefit seen in the fasting arm is secondary to carbohydrate restriction, protein restriction, or caloric restriction/being in a fasting state.

Currently, there are three studies analyzing low carbohydrate diet with a ketogenic protocol in the perioperative setting. These include a multi-institutional study out of Memorial Sloan Kettering randomizing newly diagnosed endometrial cancer patients to KD vs. standard diet in the prior to surgery (NCT03285152). A study based in France will assess a KD vs. protein restricted diet 10 days prior to surgery for breast cancer (NCT04469296). Finally, a study in Seoul, South Korea (NCT03510429) will analyze a ketogenic supplement after pancreaticobiliary cancer resections.

## Conclusion

Early results from carbohydrate restriction in both preclinical and clinical investigations are mixed. The significant increase in clinical trials examining this strategy reflects a growing interest in this approach for virtually all cancer subtypes. There is currently a paucity of evidence to suggest that a low-carbohydrate diet amongst a general population of cancer patients will improve cure rates. However, there is data to suggest efficacy when combined with some treatment modalities and in certain histologies. Additionally, strategies involving potential replacement or deferment of standard-of-care treatments with metabolic monotherapy remain limited to case studies and smaller, pilot trials (NCT03194516) (51). Though intriguing, more data is needed to validate such an approach.

Future directions of carbohydrate restriction during cancer therapy are likely to focus on hormonally sensitive tumors such as breast cancer and prostate cancer where obesity is a known risk factor. For other cancers, it may also be prudent to target patients who are metabolically unhealthy, as improving baseline metabolic health may contribute to treatment response in this population. Current studies involving epigenetics and precision medicine will further aid in the identification of patients who may benefit most from such approaches. Lastly, and perhaps most critically, a biomarker is needed to permit better comparisons of efficacy and protocol adherence within studies. One such candidate is the glucose-ketone index, which has shown utility in patients with high grade gliomas (52). Given the potential confounding impact of diet and metabolism on cancer, all studies examining cancer therapeutics or prevention would benefit from inclusion of such a biomarker.

## Author Contributions

CH contributed to the radiation, introductory, and abstract sections while also compiling information from the other authors. JC contributed to the surgery section. AK contributed to the systemic therapy sections as well as the overall structure and design of the manuscript. RK provided expertise regarding the manuscript subject matter and aided in the editing of the manuscript. All authors contributed to the article and approved the submitted version.

## Conflict of Interest

RK: Honorarium from Elsevier Inc., Elekta AB, Accuray Inc, Novocure Inc., and Viewray Inc. Research support unrelated to this study from Novocure Inc., Medtronic Inc., Blue Earth Diagnostics, GT Medical Technologies, AstraZeneca, Exelixis, and Viewray Inc. The remaining authors declare that the research was conducted in the absence of any commercial or financial relationships that could be construed as a potential conflict of interest.

## Publisher's Note

All claims expressed in this article are solely those of the authors and do not necessarily represent those of their affiliated organizations, or those of the publisher, the editors and the reviewers. Any product that may be evaluated in this article, or claim that may be made by its manufacturer, is not guaranteed or endorsed by the publisher.
